# Supplementary material for: Identification of novel cyclin-dependent kinase 4/6 inhibitors from marine natural products
Source: PLoS One. 2025 Jan 15;20(1):e0313830. doi: 10.1371/journal.pone.0313830 (PMC11734976; doi:10.1371/journal.pone.0313830)
Supplement: Attachment — Submitted filename: Response to Reviewers2.pdf [file pone.0313830.s003.pdf]

To

The Editor

PLOS ONE

Subject: Response Letter and Revised Manuscript Submission

Dear Sir,

I thank the reviewers for reviewing our paper submitted to the esteemed journal "PLOS ONE". The reviewers' feedback has been greatly appreciated and we have diligently addressed each comment in our amended article. We have highlighted in yellow color all the text that has been added or changed in the revised manuscript.

Here is our detailed reply,

Reviewer # 1:

(No Response)

Reviewer # 2:

Problem 1*:* All of my comments have been addressed. My only suggestion would be uploading high quality plots with readable legends and axis titles. Some figures are fuzzy and labels are too small to be readable.

Reply 1: As per the suggestion of the reviewer, we have uploaed the high quality figures.

Thanking you

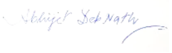

With regards

Abhijit Debnath

Asst. Professor

Noida Institute of Engineering and Technology (Pharmacy Institute), India;
